# The Role of Longevity Assurance Homolog 2/Ceramide Synthase 2 in Bladder Cancer

**DOI:** 10.3390/ijms242115668

**Published:** 2023-10-27

**Authors:** Clara Garcia-Vallicrosa, Juan M. Falcon-Perez, Felix Royo

**Affiliations:** 1 Exosomes Laboratory and Metabolomics Platform, Center for Cooperative Research in Biosciences (CIC bioGUNE), Basque Research and Technology Alliance (BRTA), 48160 Derio, Spain; cgarcia@cicbiogune.es (C.G.-V.); jfalcon@cicbiogune.es (J.M.F.-P.); 2 Centro de Investigación Biomédica en Red de Enfermedades Hepáticas Y Digestivas (CIBERehd), 28029 Madrid, Spain; 3 IKERBASQUE, Basque Foundation for Science, 48013 Bilbao, Spain

**Keywords:** tumor suppressor, cell metabolism, ceramides, biomarker, extracellular vesicles

## Abstract

The human CERS2 gene encodes a ceramide synthase enzyme, known as CERS2 (ceramide synthase 2). This protein is also known as LASS2 (LAG1 longevity assurance homolog 2) and TMSG1 (tumor metastasis-suppressor gene 1). Although previously described as a tumor suppressor for different types of cancer, such as prostate or liver cancer, it has also been observed to promote tumor growth in adenocarcinoma. In this review, we focus on the influence of CERS2 in bladder cancer (BC), approaching the existing literature about its structure and activity, as well as the miRNAs regulating its expression. From a mechanistic point of view, different explanations for the role of CERS2 as an antitumor protein have been proposed, including the production of long-chain ceramides, interaction with vacuolar ATPase, and its function as inhibitor of mitochondrial fission. In addition, we reviewed the literature specifically studying the expression of this gene in both BC and biopsy-derived tumor cell lines, complementing this with an analysis of public gene expression data and its association with disease progression. We also discuss the importance of CERS2 as a biomarker and the presence of CERS2 mRNA in extracellular vesicles isolated from urine.

## 1. Introduction

Bladder cancer (BC) is a significant cause of cancer-related deaths globally, ranking as the second-most-common reason for genitourinary cancer-related mortality [1,2]. The treatment of non-muscle invasive bladder cancer includes transurethral resection followed by chemotherapy to reduce recurrence chances, while muscle-invasive bladder cancers are associated with high rates of progression and metastasis and are usually treated via radical cystectomy if the tumor is organ-confined [3]. Unfortunately, despite treatment with radical cystectomy, BC leads to over 200,000 deaths due to metastasis [2,4]. Therefore, it remains crucial to delve into the mechanisms of BC, and in that vein, a few years ago, our group identified some transcripts enriched in extracellular vesicle (EV) preparations from the urine of BC patients that may favor early diagnosis [5]. Another essential aspect for further diagnosis and prognosis involves the categorization of malignancies. The established tumor, node, metastasis (TNM) classification system offers a classification system based on invasiveness levels. In this classification, the initial stages, denoted as Ta, are found within the mucosa, while T1 encompasses the lamina propria. Progressing from T2 signifies the invasion of muscular tissue, T3 involves the perivesical fat, and T4 extends to other organs and the abdominal cavity (discussed in [6]).

On the other hand, genome expression profiling has led to the categorization of bladder cancer into distinct molecular subtypes, some of which bear resemblance to major intrinsic subtypes identified in other types of cancers, i.e., human breast cancers. The characteristic markers associated with these two major groups mirror the expression signatures of normal basal and intermediate/luminal urothelial cell layers [7].

Among the molecular changes observed in BC tissues, the increased over-representation of the Longevity Assurance Homolog 2/Ceramide Synthase 2 genes (*LASS2/CERS2*) was particularly intriguing, given that this gene is known as a tumor suppressor, and its expression is reduced in prostate and other types of cancer, including BC [8,9]. However, various reports and datasets from cancer patients show an increase in *CERS2* in BC [10], ovarian [11], or breast cancer [12,13]. Considering these mixed findings, this review aims to uncover the role of *CERS2* in bladder cancer by combining detailed molecular information, clinical studies, and datasets from transcriptomics analyses of BC patients.

## 2. Genomic Description

*LASS2* is named after the *Lag1* gene described for *Saccharomyces cerevisiae* as Lag1 longevity assurance homolog 2 [14]. It also belongs to a mammalian family of ceramide synthases that consists of six members described so far. According to their function, they were renamed as *CERS*(1–6) [15]. Currently, the official gene symbol according to the Human Genome Organisation (HUGO) is *CERS2*, and we will maintain this nomenclature within the article for clarity. Incidentally, the same gene was cloned from a library of the human prostate cancer cell line PC-3M and described as a tumor suppressor (named Tumor Metastasis Suppressor Gene 1 (*TMSG1*)) because its expression was lower in the metastatic prostate cancer cell line PC-3M-1E8 compared to the non-metastatic PC-3M-2B4 [8,16].

Regarding their genomic features, *CERS2* gene is located on chromosome 1q21.3, and it is a complex gene. The Ensembl description of its cDNA mentions 11 exons, of which 10 are coding protein exons (Ensembl release 110 [17]). It describes 14 transcript variants, with specific alternative splicing events resulting in the exclusion of exon 8, Exon 8 of *CERS2*, corresponds to almost the entire Lag1p motif that imparts acyl chain substrate specificity to the protein, and is a part of the TRAM, Lag1p and CLN8 (TLC) catalytic domain of *CERS2* protein. This variant is more abundant in luminal breast cancer tumors [18]. Notably, the human *CERS2* gene sequence is rich in CpG elements, as revealed by CpGreport analysis, and displays a substantial presence of Alu elements [19]; the USCS Genome Browser pinpoints a single CpG site in the putative promoter region of the gene [20]. Diverse *CERS2* single nucleotide polymorphisms (SNPs) variants have been linked to diseases, as reported in the DisGeNET database [21]. Certain variants are associated with conditions such as albuminuria [22] and glomerular function [23]. Additionally, in the context of cancer malignancies, there are variants within the 3′ UTR of CerS2 that have been linked to bladder cancer [24].

Exon 1 of *CERS2* plays a pivotal role in the regulation of *CERS2* transcription. It has been indicated in the binding of Kruppel-like factor 6 (*KLF6*) and the zinc finger transcription factor, *SP1* [25]. In addition, the CerS2 promoter region harbors putative binding sites for several transcription factors, including GATA, myeloid zinc finger gene 1 (*MZF1*), two cAMP responsive element binding protein 1 (*CREB*) sites, sex-determining region Y (*SRY*), and *SP1* binding sites, which could serve as targets for G-protein coupled receptor 1 *(GPER1*) -regulated transcription factors [26]. Other transcription factors with binding sites in this region include RNA polymerase II subunit A (*POLR2A*) and chromodomain helicase DNA binding protein 2 (*CHD2*). Furthermore, the promoter region also exhibits putative binding sites for (c-) Fos, serum response factor (*SRF*), and *CREB*, all of which are known to be activated by *GPER1* [20]. Despite these findings, most reported control mechanisms governing *CERS2* are associated with miRNAs. Multiple binding sites for miRNAs have been predicted in the 3′ untranslated regions (3′ UTR) region of *CERS2*, with a comprehensive selection of both predicted and experimentally tested miRNAs provided in the corresponding section below.

## 3. Structural Properties and Protein Interactions

**Figure 1:**
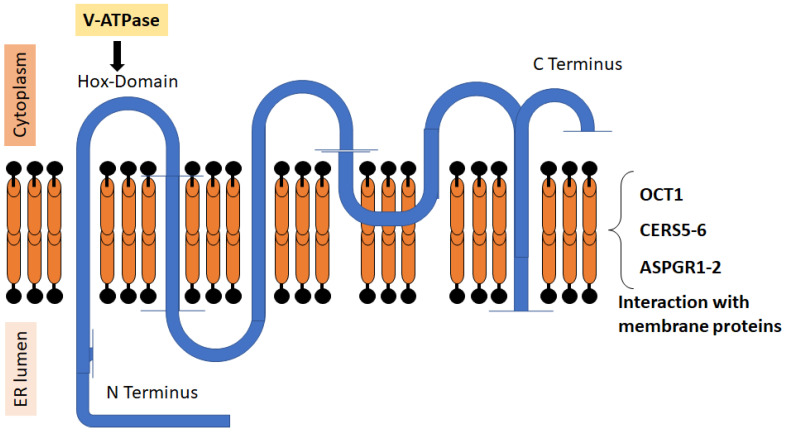
Structure of CERS2. The figure shows the relative positions of the N and C terms of the protein, the six transmembrane domains, and the HOX region that regulates v-ATPase. The protein can interact with different membrane proteins and form dimers with other CERS [20,30].

CERS2 is a protein with two main domains, a Hox domain and a TLC domain. The latter is a translocation domain [27] essential for CERS activity, and it exhibits structures required for catalysis and substrate binding [28,29]. In Figure 1, we present the different motives of the protein and its conformation around the endoplasmic reticulum (ER) membrane. In the ER, the N-terminal of CERS2 faces the ER lumen and the catalytic subunit faces the cytoplasm [30]. The reaction mediated by CERS consists in the acylation of sphinganine by fatty acyl-CoAs, which results in the formation of ceramides. Each CERS utilizes a specific subset of fatty acid chain length, and, in the case of CERS2, it specifically uses C22-C24-CoAs [19]. Among the CERS family, CERS2 is expressed in most tissues, being more abundant in the liver, lungs, heart, and kidneys [31].

Several proteins interact with CERS2. According to GST pull-down assays, it interacts with the asialoglycoprotein receptors type 1 and 2 (AGPRH1, AGPRH2), the organic cation transporter-1 (OCT1), and the proteolipid subunit of the vacuolar H+ ATPase (V-ATPase). This indicates that CERS2 acts as a membrane-associated protein [32]. Of note is the interaction with V-ATPase, which occurs directly in the Hox domain and regulates intra and extracellular pH [33]. Finally, CERS2 can also form heterodimers with CERS5 and CERS6, thereby increasing its activity [34].

## 4. Bases of Antitumoral Activity

The association of CERS2 with cancer is complex. In the beginning, it was identified as a tumor suppressor protein [16], but both roles as cancer inhibitor and promoter have been observed. It seems likely that the disequilibrium between long and very-long-chain ceramides influences cancer development (reviewed in [20]). It has been observed in vitro that the overexpression of *CERS2* increases cell proliferation and colony formation, especially after the addition of very-long chain acyl-CoAs, but simultaneously also increases apoptosis [35]. Alternative splicing of *CERS2*, specifically a form lacking the main part of the catalytic TLC domain, promotes cell proliferation and migration in luminal B subtype breast cancer cells [36]. Indeed, alternative splicing events in sphingolipid genes may contribute to the high concentration of sphingolipids-like ceramides in tumor tissues, a fact that opposes their antiproliferative role already established by in vitro and in vivo studies [37].

Specifically, in the case of BC, there is a negative correlation between the degree of expression and the malignant potential of cell lines obtained from BC [38]. Furthermore, according to studies in patients, the lack of *CERS2* expression is a poor prognostic factor associated with BC progression and invasion [39].

Different mechanisms have been proposed to explain the antitumor properties of *CERS2*. The most direct one would be deficiency in long-chain ceramides. In this regard, it has been proposed that *CERS2* promotes the apoptosis of tumor cells through ceramides [40]. The overexpression of this gene induces downregulation of BCL2 apoptosis regulator (*BCL-2*), release of cytochrome c from mitochondria, activation of procaspase-9 and procaspase-3, and cleavage of poly(ADP-ribose) polymerase 1 (PARP1) [40]. It has also been observed that very long ceramides trigger oncogenic-induced senescence induced by K-RAS [41].

On the other hand, the lack of CERS2 also results in a compensatory abundance of C14-C16 ceramides [42] and a deregulated sphingosine-1-phosphate (S1P) concentration gradient in lymphocytes and plasma [43]. Indeed, S1P is a molecule secreted into the extracellular environment that acts through S1P receptors by regulating cell–cell and cell–matrix adhesion, and thus influences cell migration, differentiation, and survival (reviewed in [44]). In BC cells, S1P receptor 1 expression has been found to be poor prognostic and is associated with the activation of TGF-β signaling [45].

Also related to ceramide concentration, the presence or absence of CERS2 has an important effect on mitochondria. As mentioned, a lack of CERS2 increases C16 ceramides. This may affect the mitochondrial respiratory chain by direct inhibition of complex IV and consequently generate oxidative stress [46]. In addition, CERS2 has been reported to block the pro-fission factor DRP1 by repressing ERK [47]. The activation of DRP1 helps the integrity of the mitochondrial membrane in the cells and confers resistance to mitochondrial apoptosis in BC cells [48].

**Figure 2:**
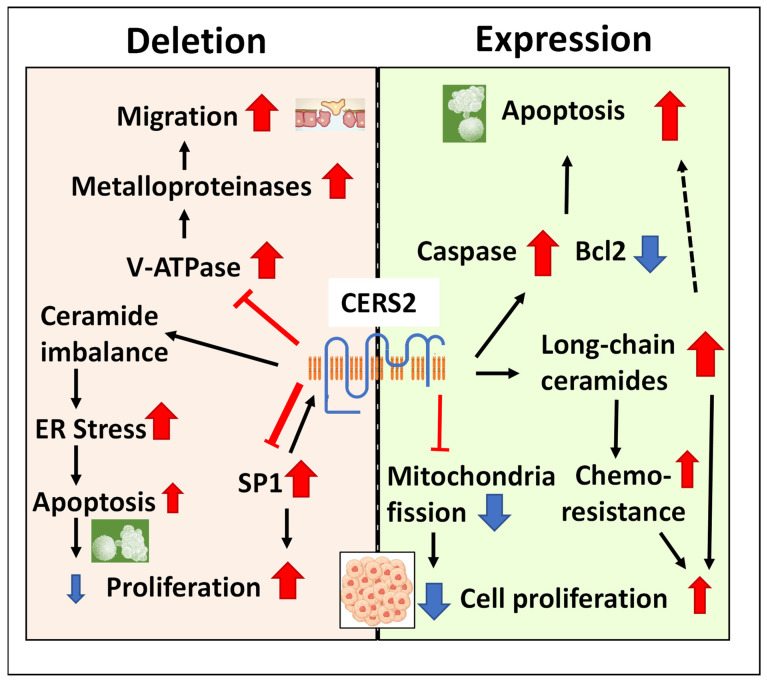
The mechanisms that explain the association of CERS2 with tumor progression. The presence of the protein maintains the cells with apoptotic capacity and avoids the mitochondrial fission characteristic of tumoral cells, although ceramide imbalance in favor of long-chain species may induce proliferation and chemoresistance. The lack of protein reduces apoptotic capacity and induces proliferation through SP1 signaling and migration by the activation of metalloproteinases, although it also may induce ER stress and apoptosis. The variable size of the arrows represents the different weight of these mechanisms in the reviewed literature.

Finally, we already mentioned that the Hox domain of CERS2 interacts directly with the C subunit of V-ATPase [33]. The silencing of CERS2 is able to increase V-ATPase activity and the extracellular hydrogen ion concentration and, in turn, the activation of secreted matrix metalloproteinase, leading to a decrease in extracellular pH that allows cell proliferation, cell survival, and cell invasion in vitro, as well as acceleration in BC growth in vivo [12]. Conversely, increase in CERS2 induces down-regulation in secreted matrix metalloproteinase-2 (MMP-2) activity [49], which explains the antimigratory effect described for this protein. Figure 2 summarizes the different antitumor mechanisms described in this section.

## 5. Regulation of CERS2 through miRNAs

MicroRNAs (miRNAs) are a class of small RNA molecules, typically consisting of 19–25 nucleotides, that play crucial roles in the post-transcriptional regulation of gene expression [50]. Since their discovery in the early 1990s, miRNAs have been found conserved through all eukaryotic cells. MiRNAs are a part of the larger family of non-coding RNAs, meaning they do not encode for proteins themselves but instead have a regulatory function. This regulatory mechanism involves the binding of miRNAs to the 3' UTRs of specific messenger RNAs (mRNAs), leading to translational repression or mRNA degradation. MiRNAs are involved in numerous cellular processes, including development, cell differentiation, proliferation, and apoptosis. Since change in miRNA levels can affect target gene expression levels, their dysregulation has been linked to various diseases, such as cancer, cardiovascular disorders, and neurodegenerative conditions [50,51,52].

For this reason, we reviewed the literature for the presence of miRNAs that regulate CERS2 expression in BC. In Table 1, we show six different miRNAs up-regulated in BC that silence CERS2 expression. For example, miR-9 expression is higher in BC tissues [53]. Furthermore, in BC cell lines transfected with miR-9, cells have increased carcinogenic characteristics and CERS2 expression is lower compared to non-transfected cells. Direct repression is likely, as there is a binding site for miR-9 in the 3′ UTR of the *CERS2* mRNA [53].

Another example is miR-20a, significantly increased in BC samples. In addition, there is a correlation between its expression and clinical stage, being higher in metastatic samples [54]. In vitro, it has also been shown that miR-20 targets *CERS2*, its expression being higher in parallel with the increasing aggressiveness of the cell lines. Similarly, in more advanced stages of the disease, miR-3622a and miR-3658 expressions are increased in bladder tumor tissue [56,57]. For both, it has also been observed that overexpression in cell lines increases proliferation and invasiveness, and *CERS2* expression is lower, both in RNA and protein. As in the previous cases, *CERS2* mRNA strand provides binding sites for these miRNAs [56,57].

In the case of miR-93, which is up-regulated in patient-derived chemo-resistant tumors, it does not directly target the 3′ UTR of *CERS2* mRNA but alters the amount of expression at the protein level [55]. This suggests that miR-93 plays a role in the chemosensitivity of BC, probably through the regulation of *CERS2*. Finally, the last miRNA reported in the literature is miR-98, which down-regulates *CERS2* mRNA and protein amounts in different BC cell lines and patient biopsies. Interestingly, miR-98 also modulates the integrity and functionality of mitochondria [48].

In addition to the miRNAs described in the literature, we would also like to mention in this review other miRNAs that could alter CERS2 expression in BC but have not yet been specifically studied. In the miRWalk database (http://mirwalk.umm.uni-heidelberg.de/ accessed 16 May 2023), we looked for predicted miRNAs interacting with CERS2, and in the mi2disease database (http://www.mir2disease.org/ accessed 18 May 2023), we found a list of possible miRNAs associated with BC. As shown in Table 1, cross-referencing both databases suggests that the miRNAs miR-125b, miR-185, miR-205, miR-222, miR-30-3p, and miR-30c may owe their association with BC to their regulatory action on *CERS2* and may be taken into account in future studies.

## 6. CERS2 Expression in BC

**Figure 3:**
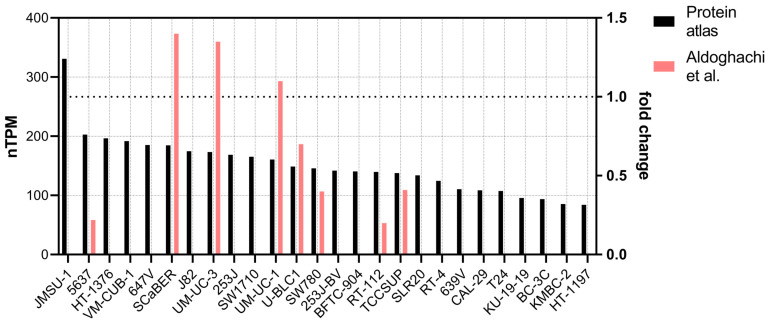
Transcript expression of CERS2 in bladder cancer cell lines. Black bars present the values obtained from The Human Protein Atlas (see text), in decreasing order. The red bars show the expression of CERS2 in some of the cell lines, normalized against the expression in UM-UC-13 cell line obtained in [3] (right axis, 1 denotes the expression of UM-UC-13).

The regulation through miRNAs points toward the silencing of the gene in tumors, and indeed, different studies have investigated the correlation between *CERS2* expression and BC progression. Regarding human bladder carcinoma cell lines, it has been described that the most malignant cells express lower amounts of *CERS2* at both the mRNA and protein levels [38,39]. However, gene expression level assessment in a panel of bladder cell lines offers wide variation. The cell lines showing higher expression of *CERS2* were a non-tumoral SV40 immortalized cell line named SVHUC1 and the cell line UMUC10, described as non-tumorigenic. On the contrary, the lowest expression was observed in UBLC1, a tumorigenic cell line. However, the second lowest expression was found in HTB4, which is described by the American type culture collection (ATCC) as non-tumorigenic in nude mice [3]. There is large agreement between that report and the classification in information obtained from The Human Protein Atlas webpage (https://www.proteinatlas.org/ accessed 13 October 2023) [58], and there is only disagreement for the bladder cell line 5637, since in this database it is recorded as having the highest CERS2 expression in spite of being quite tumorigenic according to ATCC (Figure 3).

Regarding tumoral samples, we encounter a similar situation with opposite trends described in the different studies and datasets. As in most of cell bladder cell lines, a study with 44 samples observed that *CERS2* expression decreases as the stage of malignancy increases for both protein and mRNA level [59]. This correlation has also been tested experimentally, using nude mice to study the evolution of xenografts with a highly invasive human BC cell line. Indeed, when *CERS2* was silenced in the xenografts, the resulting tumors significantly increased in volume [60]. Also supporting the tumor suppressor activity of *CERS2*, an epidemiology study based on SNPs showed that a particular substitution, which favors RNA instability and reduces transcript abundance, acts as an independent risk factor of BC susceptibility and clinical prognosis in the Chinese population [24].

However, and in spite of the previous studies supporting the idea that lower *CERS2* expression is associated with poor prognosis in BC, there are cases in which an increase in *CERS2* mRNA expression is observed early in tumor development [39]. Moreover, in silico studies and confirmation via qPCR show an increase of *CERS2* in BC [10]. The ATCG database showed that 10% of cases profiled show gene amplification for CERS2 in bladder cancer (data obtained through cBioPortal [61]), and regarding gene transcription, the expression is slightly higher in cancer samples (Log2 foldchange 0.2, with a *p* value of 0.025) among 432 samples (404 tumor vs. 28 non-tumor, data obtained through GEPIA2 [62]).

**Figure 4:**
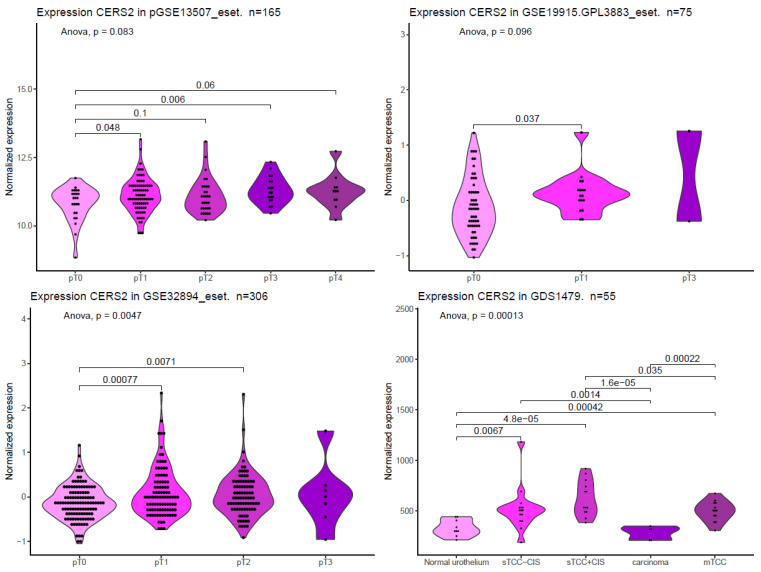
CERS2 expression in different BC datasets. These graphs were constructed from expression data available in the curatedBladderData package (in R repository), cBioPortal [61], as well as data obtained from the following articles: GSE13507_eset in [63], GSE19915.GPL3883_eset in [64], GSE32894_eset in [65], and GDS1479 in [66]. The violin graphs represent the probability density of the data, and statistical analyses were performed using one-way ANOVA, with post hoc comparisons via *t*-test, but only those comparisons with *p* value ≤ 0.1 are presented. The codification of the type of cancer was given with the data set; pTa: non-invasive papillary carcinoma, pT1: the cancer has grown into the layer of connective tissue, pT2: the cancer has penetrated the muscle layer, pT3: the cancer has crossed completely the muscle layer, and pT4: the cancer has spread into the prostate, seminal vesicles, uterus, and/or abdominal cavity. For the GDS1479 expression set, the code TCC-CIS means “superficial transitional cell carcinoma without carcinoma in situ lesion”, sTCC + CIS means “superficial transitional cell carcinoma with carcinoma in situ lesion”, and mTCC means muscle invasive carcinomas.

To complete the present review, we searched for CERS2 mRNA expression in different publicly available datasets, including GSE13507_eset [63], GSE19915.GPL3883_eset [64], GSE32894_eset [65], and GDS1479 [66]. In Figure 4, we show CERS2 mRNA expression at different stages of BC obtained from these four datasets. We can see how *CERS2* expression is significantly increased at early stages in all data sets. Interestingly, in two of the four sets, the expression is higher even in more advanced stages. As mentioned, the overexpression of CERS2 could increase proliferation. On the other hand, these results also point to differences between patients, so *CERS2* expression may be a tool for patient classification and may be used in precision medicine.

## 7. Proposed Mechanisms of Pro-Tumoral Activity

The results described in BC datasets have also been observed in a few BC cell lines in which *CERS2* is differentially overexpressed. For instance, through siRNA-mediated downregulation, a reduction in the migratory potential of the UMUC1 BC cell line has been observed [3]. For ovarian cancer, it has been observed that *CERS2*-negative tumors show significant association with longer disease-free survival and overall survival in cancer patients, and experimentally, *CERS2* overexpression in the 3AO ovarian cancer cell line promoted migration, invasion, and metastasis abilities, while *CERS2* knockdown in ES-2 and NIH:OVCAR-3 ovarian cancer cell lines had the opposite effects [11]. In the same study, the authors observed that the upregulation of the Yes1 associated protein and the transcriptional coactivator with PDZ-binding motif (*YAP/TAZ*) transcriptional regulators may be necessary for the oncogenic capacity of *CERS2* [11]. For renal cancer, however, it has been observed that increase in *CERS2*, due to its canonical activity, produces an imbalance in ceramide species associated with chemoresistance to doxorubicin. This could be reverted by inhibitors of de novo ceramide synthesis or by downregulation of *CERS2*. In this case, the clue seems to be the effect that the long-chain ceramides have over the detoxification transporter ATP binding cassette subfamily B member 1 ABCB1 regarding their recycling and functionalization [67]. In addition, it has also been proposed that the imbalance of glycosphingolipids induces severe endoplasmic reticulum stress and triggers cell apoptosis [68].

## 8. CERS2 and Extracellular Vesicles

Extracellular vesicles (EVs) are membrane-bound entities released by cells into the extracellular environment. They play an important role in intercellular communication by transporting proteins, nucleic acids, and metabolites to recipient cells, providing information about their parent cells [69]. This transfer of cargo can modulate recipient cell functions, influencing processes such as immune response regulation, cell proliferation, and tissue repair. There are three main types of EVs: exosomes, microvesicles, and apoptotic bodies [70]. Exosomes are the smallest, ranging from 30 to 150 nanometers in diameter, and are formed within the endocytic pathway. Microvesicles, also known as shedding vesicles or ectosomes, are larger, typically ranging from 100 to 1000 nanometers, and are formed through direct budding from the cell membrane. Apoptotic bodies are the largest and are released during programmed cell death. EVs have also been implicated in various diseases, including cancer, neurodegenerative disorders, and cardiovascular diseases, making them attractive targets for diagnostic and therapeutic applications [71]. EVs can be found in all body fluids, such as blood, breast milk, cerebrospinal fluid, and urine [72]. Specifically, their presence in urine has been described and well characterized for a long time [73], and their value as a diagnostic tool well documented (reviewed in [74]), including their use in BC diagnosis [75,76].

In a comparative analysis between transcripts associated with urinary EVs, the detection of CERS2 mRNA was achieved in different preparations from BC patients via both microarray and PCR technologies. In contrast, it was not detected in urinary EVs from patients with benign hyperplasia [5]. More recently, a study confirmed increase in this transcript in the urine of BC patients [10].

This result, which seems to contradict the role of CERS2 as a tumor suppressor, is nevertheless in agreement with the results observed in different gene expression data sets in BC biopsies (Figure 4). However, it should be noted that the presence of mRNA in EVs does not necessarily imply increased gene or protein expression in tumor tissue. In fact, the fate of some miRNA-silenced mRNAs may be their release through EVs by the binding of a zip code to a central domain in the stem–loop structure. For example, miR-1289 binds directly to this zip code of its target mRNA and orchestrates its transfer to microvesicles [77].

Regarding the possible role of circulating CERS2 in EVs as a biomarker, there are several reports on the presence of the protein and mRNA in EV preparations registered in Vesiclepedia archives (http://microvesicles.org/ accessed 6 June of 2023) isolated from different tumor tissues such as glioblastoma, colorectal, breast, or brain cancer. Interestingly, dendritically derived EVs loaded with CERS2 had an anti-apoptotic effect in neurons in and age-driven inflammatory context [78]. Moreover, ceramides synthesized by CERS2 are also released and transported by microvesicles [79] and the lack of function of the protein in some tumors probably modifies the composition of their EVs. Therefore, both the presence of circulating *CERS2* mRNA or protein as cargo in EVs, as well as the composition of these EVs, have great potential for establishing not only the diagnosis but also the prognosis of patients with BC.

## 9. CERS2 as a Therapeutical Target

Regarding chemotherapy, the standard approach for treating patients with advanced urothelial cancer is the so-called methotrexate, vinblastine, doxorubicin, and cisplatin regimen. Unfortunately, due to high toxicity and suboptimal responses, there has been a need to explore newer treatment combinations. For example, the regimen using gemcitabine and cisplatin was found to yield similar survival outcomes with fewer side effects. These chemotherapies demonstrate high initial response rates, with a median survival of 15 months (reviewed in [80]). A recent approach is based on immune checkpoint inhibitors, which are approved as a second-line treatment for patients who experience progression after initial cystatin treatment [81]. These drugs are effective in tumor cells that express PD-L1 proteins, blocking the binding of the ligand to the associated checkpoint receptor. In this context, it is worth mentioning that basal subtypes of bladder cancer, characterized by p63 activation, squamous differentiation, and a more aggressive disease, also express higher levels of immune checkpoint ligands, specifically programmed-death ligand 1 (PD-L1), compared to luminal tumors [82]. Hence, stratifying bladder cancer is essential for guiding therapeutic decisions.

To address this objective, the integration of genetic and molecular information can provide valuable guidance for treatment decisions in newly diagnosed bladder cancer (BC) patients. Considering the dual role observed for CERS2, this gene emerges as a promising candidate for molecular markers to inform therapy decisions. CERS2 plays a pivotal role in multiple survival mechanisms, and manipulating its expression and activity can significantly affect cell proliferation and tumor progression. Numerous studies have delved into the molecular implications of this gene’s involvement. As a key player in the synthesis of long ceramides, CERS2’s role in the context of chemotherapy appears to promote chemoresistance. The upregulation of *CERS2* induced by treatment with C2-ceramide increases the protein levels of CERS2, thereby modulating sphingolipid metabolism to favor the conversion of C2-ceramide into pro-survival sphingolipids in hepatocarcinoma cells. This mechanism is associated with autophagy and a reversible senescence phenotype, ultimately contributing to C2-ceramide resistance in these cells. However, co-treatment with polyphenols downregulated the protein level of CERS2 and increased oxidative and endoplasmic reticulum stress, leading hepatocarcinoma cells toward apoptosis [83]. In the context of renal malignancies, the downregulation of *CERS2* reduces doxorubicin chemoresistance. This action is mediated by the reduction in the protein amount and plasma membrane localization of ABCB1, a transmembrane transporter that extrudes both proapoptotic substrates and drugs out of the cell. Again, this effect is caused by an imbalance in ceramide species [67].

On the contrary, in the case of some solid tumors, the activity of proteins such as BCL2, with the capacity to bind to and inhibit CERS2, takes the opposite course. For instance, in glioblastoma tumor cells with high expression of BCL2 like 13 (*BCL2L13*), CERS2 activity is blocked, resulting in the prevention of apoptosis in response to both conventional and targeted therapies. It is noteworthy that pharmacological inhibition of BCL2L13 may offer the potential to increase proapoptotic ceramide levels in cancers by restoring CERS2 activity [84]. In the same direction, treating cells with diterpenoids that reduce BC2 and increase CERS2 activity also increases apoptosis in glioma cells [68]. As we have observed, in different contexts, the activity of CERS2 can either promote apoptosis or inhibit it, depending on various factors, highlighting its versatile role in cancer biology.

## 10. Conclusions

In this review, we mentioned several studies that underscore the important implications that ceramides and their synthesis have in cell physiology and pathophysiology and taken together suggest that alteration of CERS2 functionality influences the development of cancer. First, the literature shows that CERS2 is necessary for the correct mechanism of apoptosis. Likewise, many studies in patients highlight that the loss of expression of this gene represents a poor prognosis in the evolution of BC. Regarding therapy, the silencing of BCL2 family proteins increases CERS2 activity and apoptosis. However, not all the studies move in the same direction, and some data show that reduction in CERS2 activity favors ER stress and apoptosis. The disparity in the results highlights the necessity of improving cancer sub-classification based on molecular markers. It is worth emphasizing that when examining BC subtypes based on their transcriptional signatures, similarities emerge between different types of tumors. For instance, parallels have been observed between breast cancer luminal tumors and luminal BCs, as well as between basal muscular invasive BC and squamous tumors originating in the head and neck region [85]. However, CERS2 is not one of the genes that distinguishes both types of tumors. In conclusion, different expression data sets and clinical samples show upregulation of the gene in early stages of tumor development, which is consistent with the role found for CERS2 in other malignancies. In the case of ceramides, as with other essential biomolecules, loss of tight cellular regulation results in pathological situations that favor tumor progression. Finally, we would like to emphasize that the presence of CERS2, both mRNA and protein, in urinary EVs constitutes a potential tool for the diagnosis and prognosis of BC.

## Figures and Tables

**Table 1:** miRNAs found upregulated in BC patients or cell lines, able to downregulate CERS2 expression.

miRNA	In Vitro and In Vivo Validated	Proposed for Validation	Reference
miR-9	yes		[53]
miR-20a	yes		[54]
miR-93	yes		[55]
miR-98	yes		[48]
miR-3622a	yes		[56]
miR-3658	yes		[57]
miR-30-p		yes	Cross reference of the databases miRWalk and mir2disease (see text)
miR-30c		yes	
miR-125b		yes	
miR-185		yes	
miR-205		yes	
miR-222		yes	

## Data Availability

Not applicable.
